# Health Care Utilisation and Attitudes towards Health Care in Subjects Reporting Environmental Annoyance from Electricity and Chemicals

**DOI:** 10.1155/2009/106389

**Published:** 2009-04-14

**Authors:** Frida Eek, Juan Merlo, Ulf Gerdtham, Thor Lithman

**Affiliations:** ^1^Department of Occupational and Environmental Medicine, Lund University, SE 221 85 Lund, Sweden; ^2^Department of Social Medicine, Lund University, SE 205 02 Malmö, Sweden; ^3^Department of Health, The Health Centre of The County Council, Scania Region, SE 221 00 Lund, Sweden

## Abstract

Environmentally intolerant persons report decreased self-rated health and daily functioning. However, it remains unclear whether this condition also results in increased health care costs. The aim of this study was to describe the health care consumption and attitudes towards health care in subjects presenting subjective environmental annoyance in relation to the general population, as well as to a group with a well-known disorder as treated hypertension (HT). *Methods*. Postal questionnaire (*n* = 13 604) and record linkage with population-based register on health care costs. *Results*. Despite significantly lower subjective well being and health than both the general population and HT group, the environmentally annoyed subjects had lower health care costs than the hypertension group. In contrast to the hypertension group, the environmentally annoyed subjects expressed more negative attitudes toward the health care than the general population. *Conclusions*. Despite their impaired subjective health and functional capacity, health care utilisation costs were not much increased for the environmentally annoyed group. This may partly depend on negative attitudes towards the health care in this group.

## 1. Introduction

Through the years several so-called “modern diagnoses” have succeeded each other. Subjective health complaints attributed to the pressure of “modern life” have been described in the medical literature since the 19th century [1]. The similarities between these different syndromes have often been larger than the differences. The most obvious difference between the syndromes has been the attribution of the symptoms.

Even though some of the diagnoses have some specific components, most of the symptoms are shared among almost all of these conditions [2]. Another similarity is that there are no objective medical tests to confirm these diagnoses; instead, the diagnoses are based on reporting of symptoms [3–6]. There have been many suggestions for a common term for these syndromes, but as yet no agreement has been reached regarding a suitable term. “Medically unexplained symptoms (MUSs),” “unexplained clinical conditions,” “functional somatic syndromes,” “somatisation” and “subjective health complaints,” “somatoform disorders,” “fashionable diagnoses,” “functional symptoms,” and “chronic multisymptom illness” are some of the terms that have been used to describe the condition of somatic complaints not explained by any known somatic disease. The present study focuses on one form of the so-called medically unexplained symptoms (MUSs), namely symptoms or annoyance related to electricity and/or chemicals and smells—environmental illness or intolerance. When the concern about illness attributed to electricity was first raised, the terms “electric allergy” and “hypersensitivity to electricity” were commonly used. Use of these terms is not recommended; however, since they imply an established aversive physiological effect of EMFs. No such effects have yet been proven [7], which is why the terms should preferably not be used. “Sensitivity to electricity” is another frequently used term. Since it does not directly imply allergic or “hypersensitivity” reactions, it is at least a little more appropriate. This term could be viewed as a correlate to the more established term “multiple chemical sensitivity (MCS)”. However, during the years other terms that probably better describe the phenomenon in question have been suggested. In 1996, the term “idiopathic environmental intolerance (IEI)” was suggested to replace “MCS” [8]. “Idiopathic environmental intolerance” covers subjective illness attributed to a variety of environmental factors normally tolerated by the majority of people. Moreover, the symptoms should not be explained by any known medical or psychiatric disorder [8–11]. This term is preferable, since it does not make any unsupported judgement about the cause of the symptoms or illness [10]. In 2004, it was added that the term IEI should not be used as a medical diagnosis, because of the lack of scientific basis to link the IEI symptoms to EMF exposure [12]. The term “IEI” includes both MCS and sensitivity to electricity [11].

The present paper examines a somewhat less pronounced form of IEI, which we call “environmental annoyance”. Such annoyance from environmental factors (i.e., “visual display units,” “fluorescent tube light,” “other electrical equipment,” “chemicals” and “other smells”) has been found to be rather common in the population. A previous study showed that 28% of a Swedish population reported that they had been a little annoyed, and 6% much annoyed, by at least one of these environmental factors during the past two weeks [13]. Subjects reporting annoyance from environmental factors were found among most groups in society, but it was more common among women, immigrants, unemployed, disability pensioners, and students. Environmentally annoyed subjects presented low self rated health, and difficulties to perform every-day duties. Subjects experiencing annoyance from *both* electrical and chemical factors (2–4%) was found to be the most affected regarding subjective health and functioning, and also experienced higher degree of every day stress and subjective health complaints [13–15]. Apart from these studies, “environmentally annoyed” populations (by this definition) have not been studied much so far. In contrast, symptomatic IEI patients have been found to be more anxious, stressed, and depressed than people without IEI [16].

Health care utilisation in subjects suffering from environmental intolerance related to electrical factors has scarcely been examined [17, 18]. However, military personnel reporting ideopatic environmental intolerance (IEI)/multiple chemical intolerance (MCS) had higher out-patient rates of physician visits, emergency ward visits, and in-patient hospital stays than did military personnel without IEI/MCS [19]. One study showed an average of 23 health care visits per year for IEI subjects [20]. McGlone et al. [21] found increased health care utilisation in 24 persons reporting environmental illness, compared with 48 controls. In another study, among subjects reporting hypersensitivity to common chemicals 45% reported that they had received medical treatment [22]. However, the study revealed neither what kind of medical treatment was used, nor the extent of the treatments. A study of 917 MCS subjects revealed that the participants had consulted a mean of twelve health care providers [23]. By contrast, only a few of subjects experiencing symptoms in connection with mobile telephone use reported that they had consulted a physician or been on sick leave because of the symptoms [18]. These two studies were based on self-reports on health care and treatments. A study examining psychiatric symptoms and medical utilisation in primary care patients revealed that patients identified as depressed and/or anxious, that is, presenting symptoms that have been associated also with IEI-patients, reported significantly increased medical utilisation compared with patients without depression or anxiety. However, this was not confirmed by the hospital's computerised record system, which revealed no significant differences between the groups with and without depression or anxiety [24]. These results indicate that self-reports of health care utilisation may not always be reliable, especially when examining subjects who may be depressed or anxious.

MUS and somatisation have been associated with increased health care utilisation [25–32]. MUS have also been associated with frequent primary care seeking [27, 33]. “Modern health worries” have been found to be associated with medical care utilisation, mainly with alternative health practitioners [34].

Patients with MUS have been experienced as difficult to manage by general practitioners [35], which may impair the relation between doctor and patient, and originate feelings of frustration for the general practitioner [36]. Patients with medically unexplained symptoms have also reported frustrating experiences of their contact with doctors [37, 38]. The question as to what causes environmental annoyance is quite controversial, and the opinion of patients and doctors may be divergent, which may contribute to the reluctance to seek and give medical care. There may be reasons to believe that meeting patients expressing annoyance from electricity and smells may give rise to negative experiences not only for the doctor but also for the patient. These aspects might condition a reduced access to health care resources for these patients. The question that rises is how subjects annoyed by electricity and chemicals utilize the medical care system. Do they get medical care or treatment for their problems? Since these subjects present decreased self-rated health, in combination with the facts that MUS in general have been associated with increased health care utilisation, it might exist a preconception that these subjects also originate high health care costs.

### 1.1. Aims

The aim of this study was on the one hand to describe health, health care consumption, and subjective experience of health care in subjects annoyed by both electricity and smells and, on the other, to rank this group in relation to the general population, and a group with a well-known disorder as treated hypertension.

## 2. Methods

### 2.1. Participants

The Health Survey Scania 2000 (HSS-2000) was a postal self-administered questionnaire sent out between November 1999 and April 2000 to a random sample of 23 437 individuals born from 1919 to 1981 living in Scania. In total, 13 604 subjects (58%) participated. The 13 604 respondents have been compared with the entire population in same ages in Scania (*n* = 850 476) and found to be fairly representative regarding age, gender, and health care utilisation [39].

The Ethical Committee at the Medical Faculty of Lund University approved (LU 179-99) the study proposal of The HSS-2000, and all of the participants received written information about the survey.

### 2.2. Subject Grouping

Five questions regarding environmental annoyance were included in the survey, to assess the prevalence of annoyance related to electrical and chemical factors in the population. These questions read: “Did you during the past 14 days experience annoyance that you associate with (1) fluorescent tube lighting/(2) visual display units/(3) other electrical equipment/(4) breathing air that smells of chemicals/(5) other smells and if so, how much annoyance did that cause you?” with possible responses “No,” “Yes, some” or “Yes, very much”. In this study, we chose to focus on the subjects who reported annoyance from both any electrical factor and chemicals or smells, at least one of the factors as being much annoying (*n* = 315).

Treated hypertension (*n* = 1373) was defined as use of antihypertensive medication, based on an affirmative answer to the question “Have you during the last year used medicine, which was bought at the pharmacy…?”… “For the treatment of high blood pressure”.

Subjects in the electrical and chemical annoyed group who were also treated for hypertension (*n* = 20), were excluded from the analyses, as well as subjects who did not leave information on neither HT medication nor environmental annoyance (*n* = 29). The final number of subjects in each group is presented in Table 1.

### 2.3. Outcome Measures

#### 2.3.1. Health Care Utilisation

Health care utilisation was the main outcome measure in our study, and it was approximated by information on number of visits to general practitioners and direct health care costs in SEK. This information was provided to us by the health authorities in the county which calculated it following the same standard procedures all over the county [40].

A ten-digit civic registration, assigned to each individual in Sweden for their lifetime, is recorded at the county of Scania in the Patient Administrative System (PASIS) for public care and PRIVA for private care, and also in the Health Survey 2000, and was used for record linkage. The Regional Office at the Scania County Council did the tabulation preserving the anonymity of the subjects. Health care costs were related to health care visits for the year 1999. The total cost for each nursing ward, clinic, and hospital was broken down into individual in-patient nursing contact. The Diagnosis-Related Groups (DRG) system is currently used in many countries as a proxy for the cost of in-patient care [41]. When the actual cost for each visit was unknown, DRG codes were used to calculate costs for each visit. Visits that had no DRG code were given the mean amount of DRG points for the clinic. If the clinic did not use DRG codes, the cost was divided among days when medical care was provided. Out-patient costs were calculated directly through a Patient Cost System, where every contact received a specific cost for the actual use of resources. The costs included correspond to what in health economics is referred to as direct costs (i.e., out-patient and in-patient care including surgery, intensive care, diagnostic services, and drugs). Cost of drugs included concerned only what was consumed by the patients during in-patient care. Costs of drugs in out-patient care are not available on the specific patient level.

#### 2.3.2. Additional Variables and Outcome Measures

The survey also included among other items, information on self-reported health (SRH-7) [42], mental well-being (General Health Questionnaire [GHQ-12], [43]), medication use including alternative medication, and experiences of the health care system, later dichotomized with the negative response as outcome; such as “do you consider yourself having been in need of, but not sought, medical care” (i.e., “unsatisfied health care needs”), “Was the medical staff open to your wishes/needs” (i.e., “neglected health care needs”), “Did the doctor treat you with kindness and respect on your latest visit” (i.e., “nonrespected health care needs”), and “What is your experience of the health care in your local authority” (i.e., “mostly negative experience of health care”).

### 2.4. Statistical Analysis

For statistical analysis, the SPSS computer software version 11.0 was used. Odds ratios (ORs) and 95% confidence intervals (95% CI) were estimated by use of conditional logistic regression adjusting for age and gender. Means of health care utilisation costs, number of visits, and subjective well-being, were calculated through univariate analysis of variance (ANOVA). Since the cost data was not normally distributed, the Kruskal Wallis *H*-test was used to test the null hypothesis of nondifferences in health-care consumption costs between groups. Pairwise comparisons between groups were performed through the Mann-Whitney *U*-test. A *P*-value below .05 was considered statistically significant. Mean costs and 95% confidence intervals are also presented.

## 3. Results

### 3.1. Subjective Health Status

The groups differed in their ratings of subjective physical and mental well-being (*P*'s < .001). The chemical-electrical annoyed group reported lower SRH-7 scores than the hypertension (HT) group (post hoc: −0.70 [−0.87; −0.53]) and the general population (post hoc: −1.02 [−1.18; −0.87]). The HT group also differed from the general population (post hoc: −0.33 [−0.40; −0.24]). The chemical-electrical annoyed group also presented higher GHQ-scores compared to the HT group (post hoc: 0.26 [0.21; 0.31]) and the referents (post hoc: 0.32 [0.27; 0.36]). The HT-group also differed from general population (post hoc: 0.06 [0.04; 0.08]) (see Table 1).

### 3.2. Health-Care Costs

Regarding mean health care costs, the electrical-chemical annoyed groups presented similar mean total health care costs as the general population, with overlapping the 95% confidence intervals. The HT group showed significantly higher mean costs than the general population. The nonparametric tests showed significantly higher median costs for both the HT and the chemical-electrical annoyed group, compared to general population (*P*'s < .001). The HT group also had significantly higher median cost than the electrical-chemical annoyed group (*P* < .001) (see Table 1). Regarding the primary health care cost, both the environmentally annoyed group and the HT group had significantly increased mean and median costs compared to general population (*P*'s < .001). The HT group also had significantly higher median cost than the electrical-chemical group (*P* < .001).

The electrical-chemical annoyed group and the HT group had significantly more visits to general practitioners than referents, both regarding mean and median number of visits (*P*'s < .003) (see Table 1.) The HT group also had significantly higher median number of visits than the electrical-chemical annoyed group (*P* < .001). Mean and median costs and number of visits are presented in Table 1.

Compared with the reference group, the odds ratio of having had some health care cost preceding year was 1.6 (1.2; 2.2) in subjects with electrical and chemical annoyance (adjusted for age and gender). This odds ratio was 5.9 (4.4; 7.8) in patients treated for hypertension compared with the general population (not shown in tables).

#### 3.2.1. Experiences of Health Care

The odds ratios of reporting unfulfilled health care needs, of considering that the medical system neglected one's needs and of not being treated with respect, were increased in those reporting both electrical and chemical annoyance compared to referents (*P*'s < .004). The environmentally annoyed group had also increased probability of having “mostly negative experience” of the health care in their municipality (*P* < .001). The HT group did not differ from the general population on any of these variables. The electrical and chemical annoyed group, but not the HT group, had significantly increased OR to have used alternative medication (*P* < .001) (see Table 2).

## 4. Discussion

Even though the electrical-chemical annoyed group was the group with the lowest subjective health and the least well-being, health care utilisation costs were not remarkably high as compared with the general population or with a patient group with a relatively common medical condition as treated hypertension. Rather, results suggest that electrical-chemical annoyed subjects do not get, or seek, the health care that could be expected according to their reduced subjective health. This last aspect could presumably be linked to their reported negative experiences with the health care system. These negative attitudes and experiences may probably origin in the fact that there is a great discrepancy in what treatment that is being offered in traditional medicine regarding problems associated with environmental intolerance, and what is usually preferred by the persons experiencing these problems.

Since the distribution of costs is extremely skewed, mean costs and ANOVAs are not the most appropriate way for analysing differences between groups. When looking at median costs, even though the difference between the electrical-chemical annoyed group and the general population is statistically significant, it is not great in absolute terms. Moreover, the HT group presented significantly higher median costs than the electrical-chemical annoyed group, despite significantly better subjective well-being.

A direct comparison between the HT group and the electrical-chemical annoyed group might appear inappropriate due to the different nature of these two medical conditions. The HT group is also much older than the other groups. But, although adjustment for age decreases the difference between the HT group and the other groups in mean health care costs, the HT group still cost significantly more than both the other groups after adjustment for age and gender. This adjustment was not possible to perform regarding median cost, but theoretically, an adjustment for age difference would possibly have a quite similar effect, that is, reduce the present differences, but not abolish it. This effect was seen in analyses of ranked or log-transformed cost data. The differences remained even after age and gender adjustments; hence, the HT group still cost significantly more than the other groups. However, we want to stress that the aims of our comparisons were strictly to describe and relate the heath and health care utilisation of the electrical-chemical annoyed group to the general population and to the HT patients. We did not investigate any causal hypothesis since those groups are not comparable in this perspective.

The nature of environmental sensitivity gives rise to expectations of increased cost [19, 25–31, 34]. Medically unexplained symptoms have been associated with “frequent attenders” in primary care [27, 33]. “Frequent attenders” were defined as the top 5% of the out-patient attenders, by the number of appointments [33], which in our material would be >5 visits. So, despite a slightly higher number of visits to GP's compared with the reference group, the environmentally annoyed subjects are far from being frequent attenders. Nonetheless, subjects in this group did not feel well, and they often reported to have been in need of, but not having sought, medical care.

So, why did the environmentally annoyed subjects not seek medical care? One explanation may be their negative experiences of health care. These experiences may be a result of various factors, and negative or frustrating experiences of health care providers have also previously been reported in other studies on MUS patients [37, 38]. Today, there is no generally accepted treatment for environmental intolerance. This could be one explanation why the environmentally annoyed subjects did not seek medical care to a greater extent. Even if they did seek care, this contact would probably not have resulted in continuous care because of the lack of treatment options for environmental intolerance. The results also contradict that the reduced health status in the environmentally annoyed groups would be explained by other sicknesses. If the reduction in health would depend on other sicknesses, more familiar to physicians than environmental illness, one would expect them to have been treated for these conditions, which would have led to evidently increased costs.

In a previous follow-up study, 50 subjects with perceived hypersensitivity to electricity revealed that 38% had used “complementary therapies” [44]. In the present study, almost half of the subjects in the environmentally annoyed group had used alternative medication. Even though alternative medication was commonly used also among the referent population and the HT group, it was even more common in the environmentally annoyed group. This could indicate a tendency to seek alternative ways to handle with experienced symptoms or health related problems. This tendency could be a result of dissatisfying experiences with the traditional health care. We did not have information on alternative care except from medication; hence, we do not know whether they also seek alternative care to a higher extent. In a previous study, only a few of subjects experiencing symptoms in connection with mobile phone use reported that they had consulted a physician or been on sick leave because of the symptoms [18]. In one study on subjects suffering from “hypersensitivity” to electricity, fewer of the afflicted subjects used conventional medication than the nonafflicted [17]. However, in our present study we have no results supporting that the increased use of alternative medication excluded use of more traditional medication.

A model for health care utilisation and somatisation was presented by Ciechanowski et al. [45]. This model was based on the attachment theory, showing that the attachment style was associated not only with symptom reporting but also with health care utilisation. Data from that study showed that subjects with a “fearful” attachment style had the lowest primary care cost, despite having just as many symptoms as subjects with “preoccupied” attachment style, which had the highest costs. Our study does not examine attachment style, but this is one factor that may influence health care utilisation, irrespective of the number of symptoms reported. The experience of, or attitudes towards, health care may also be linked to attachment style. According to Bowlby, who first developed attachment theory, individuals internalise earlier experiences of caregivers, forming cognitive schemas of relationships that may influence not only whether they perceive themselves as worthy of care, but also whether others can be trusted to provide care [45]. These “models of other” may influence the interactions the individual has with others, and also form the interpretations of these interactions throughout life. What cognitive schemas that are formed, is suggested to be depending on the individuals' attachment style. A “secure” or “preoccupied” attachment style is associated with larger trust or dependence on others, while a “dismissing” or “fearful” attachment style is associated with “compulsory self-reliance” or approach avoidance behaviour. Hence, also the attitudes of health care found among the environmentally annoyed persons in the present study may be related to these attachment styles, which are also associated with decreased health care utilisation regardless of symptom severity. Both the negative experiences and attitudes, and the not much increased health care utilisation hence indicate the presence of a fearful (or perhaps dismissing) attachment style among the environmentally annoyed subjects.

### 4.1. Study Limitations

In the present study, we analysed direct health care cost only, as information on costs for sick-leave, unemployment, and so forth were not available. These indirectly associated costs may be increased and deserve further studies. Also costs for alternative treatments would be highly relevant to examine in the environmentally annoyed group, as there are reasons to expect these costs to be increased which is also supported by the findings of increased OR of use of alternative medication as measured by the direct question in the survey. However, the aim with this study was to analyse utilisation of and attitudes towards “traditional” medicine, which was what we did.

Outpatient medication costs are not included in the health care costs which leads to an underestimation of health care cost among patients treated for hypertension, and support our conclusions regarding the electrical-chemical annoyed group. A strength of the study is the access to record based data on health care utilisation, which reduces the reliability problems associated with self-reported health care [24].

Although the response rate was no higher than 59%, the analysis of representativity indicates that selection bias should not be a substantial problem, at least not in relation to information on health care consumption [39].

We performed extra calculations using only subjects reporting *no* annoyance to any of the five factors as control group but the results were similar. These extra analyses suggest that our main results regarding health care utilisation should not depend on misclassification bias caused by a lack of clear cut off regarding environmental annoyance between the annoyed group and the population.

In the present study, we analysed direct health care cost only as information on costs for sick-leave, unemployment, and so forth were not available. These indirectly associated costs may be increased and deserve further studies.

## 5. Conclusions

In conclusion, despite their impaired subjective health and functional capacity, health care utilisation costs were not much increased for the environmentally annoyed group. Negative experiences in their contact with the health care system and perhaps other factors not investigated in the present study may contribute to the possibility that these patients do not get, or seek, the health care expected according to their reduced subjective health.

## Figures and Tables

**Table 1:** Characteristics of the population in relation to environmental annoyance and treatment of hypertension.

	Electrical and chemical annoyance	Treated hypertension	Ref.
Number of subjects	295	1353	11907
Mean age	43	64	47
Women (%)	66	53	54
Cost > 0 SEK (%)	84	96	77
Having been in need of, but not	49	17	20
having sought, medical care (%)			
Medical staff neglected	25	4	7
my wishes/needs (%)			
Not treated with respect	6	1	3
from doctor on last visit (%)			
Mostly negative experience (%)	19	4	6
Use of alternative medication (%)	49	33	28
General Health Questionnaire	2.26 (2.22–2.31)	2.01 (1.98–2.03)	1.95 (1.94–1.95)
mean^(a)^ score (95% CI)			
Self-rated health score,	4.13 (3.98–4.29)	4.83 (4.76–4.91)	5.16 (5.13–5.18)
mean^(a)^ (95% CI) SEK			
Mean total cost (95%	6 658 (3 182–10 134)	14 424 (12 807–16 040)	7 890 (7 346–8 435)
Confidence Interval) SEK			
Mean^(a)^ total cost	7 888 (4 417–11 358)	11 698 (10 024–13 372)	8 216 (7 664–8 767)
(95% Confidence Interval) SEK			
Median total cost SEK	2519	4329	1628
Mean^(a)^ GP cost	1371 (1195–1546)	1594 (1510–1679)	963 (935–991)
(95% Confidence Interval)			
Median GP cost SEK	790	1 185	395
Mean^(a)^ (95% CI)	1.9 (1.6–2.1)	2.4 (2.3–2.5)	1.4 (1.4–1.5)
number of visits to GP			

^(a)^Age and gender adjusted.

**Table 2:** Odds ratio (95% CI) of experiencing unsatisfied, neglected, and nonrespected health care needs, having mostly negative experience of health care, and of having used alternative medication.

	Electrical and chemical annoyance	Treated hypertension
	OR (95% CI)	OR (95% CI)
“Unsatisfied health care needs”	3.5 (2.8–4.5)	1.0 (0.8–1.2)
“Neglected health care needs”	3.8 (2.9–5.0)	1.1 (0.8–1.5)
“Nonrespected health care needs”	2.1 (1.3–3.4)	0.7 (0.4–1.3)
“Mostly negative experience of health care”	3.3 (2.5–4.5)	1.3 (0.9–1.7)
Having used alternative medication	2.4 (1.8–3.0)	1.1 (1.0–1.3)

Age and gender adjusted.
